# An Overview on Antimicrobial Potential of Edible Terrestrial Plants and Marine Macroalgae Rhodophyta and Chlorophyta Extracts

**DOI:** 10.3390/md21030163

**Published:** 2023-02-28

**Authors:** Silvia Lomartire, Ana M. M. Gonçalves

**Affiliations:** 1 University of Coimbra, MARE—Marine and Environmental Sciences Centre/ARNET-Aquatic Research Network, Department of Life Sciences, Calçada Martim de Freitas, 3000-456 Coimbra, Portugal; 2 Department of Biology, CESAM—Centre for Environmental and Marine Studies, University of Aveiro, 3810-193 Aveiro, Portugal

**Keywords:** bioactive compounds, Rhodophyta, Chlorophyta, antimicrobial resistance, natural antibiotics, seaweeds

## Abstract

Antibiotics are used to prevent and treat bacterial infections. After a prolonged use of antibiotics, it may happen that bacteria adapt to their presence, developing antibiotic resistance and bringing up health complications. Nowadays, antibiotic resistance is one of the biggest threats to global health and food security; therefore, scientists have been searching for new classes of antibiotic compounds which naturally express antimicrobial activity. In recent decades, research has been focused on the extraction of plant compounds to treat microbial infections. Plants are potential sources of biological compounds that express several biological functions beneficial for our organism, including antimicrobial activity. The high variety of compounds of natural origin makes it possible to have a great bioavailability of antibacterial molecules to prevent different infections. The antimicrobial activity of marine plants, also called seaweeds or macroalgae, for both Gram-positive and Gram-negative, and several other strains infective for humans, has been proven. The present review presents research focused on the extraction of antimicrobial compounds from red and green macroalgae (domain Eukarya, kingdom Plantae). Nevertheless, further research is needed to verify the action of macroalgae compounds against bacteria in vitro and in vivo, to be involved in the production of safe and novel antibiotics.

## 1. Introduction

The advent of antibiotic resistance, which has become a barrier in the treatment of numerous types of diseases caused by bacterial infections, has made infectious diseases one of the global leading causes of death cases [1,2]. Antibiotic resistance occurs for different reasons: bacteria neutralize antibiotics by pumping them out of cells or changing the structure of their cell walls to minimize the entering of the drug within bacterial cells [3]. Antibiotic resistance is due especially to the overuse and misuse of antibiotics in human and animal health and the lack of development of new antibiotics that minimize resistance.

The research for new bioactive compounds is one of the solutions to overcome this problem [4,5]. Natural products are a source of secondary metabolites with a variety of structures and bioactivity. Moreover, natural products have high availability and may exhibit similar properties to synthetic antibiotics but, additionally, they could prevent drug resistance [4,6]. Originally, a limited group of marine species, including sponges, mollusks, tunicates, and macroalgae, were the main targets of marine wildlife exploitation for natural bioactive compounds [7]. These were demonstrated to form a wide variety of unusual molecular structures, including prostaglandins, polyketides, and halogenated terpenes that present interesting biological activities [8,9,10]. This variety of bioactive structures is thought to be a component of these species’ defense, survival, and predation strategies [11]. Marine natural products are examples of novel and varied chemotypes that can be used as models to find and create therapeutic medicines. These genetically encoded molecules are typically highly complex and can be challenging to replicate in laboratories. Moreover, most of the time, chemicals are limited due to the low yield isolated from the source organism, but thanks to sophisticated NMR technologies and dereplication techniques they are able to reproduce more bioactive compounds of interest [12].

Penicillin was discovered by Alexander Fleming in the 1920s from a culture of the fungus *Penicillium notatum* [13]. Out of the 162 antibacterial agents approved by the U.S. Food and Drug Administration from 1981 to 2019, about 50% are or derive from natural sources [14], and most compounds have a microbial source rather than a plant source [15].

Nevertheless, in most in vitro studies carried out in recent years, the antibacterial properties of plant extracts have been confirmed [16,17,18,19,20].

It is also important to develop further studies regarding the combination of natural products with common antibiotics on multidrug-resistant bacteria. Nascimento et al. [21] showed the beneficial effects of mixing the antibiotic methicillin with natural extracts. The authors investigated extracts of clove, jambolan, pomegranate, and thyme. The synergistic effect of antibiotic compounds prevented the development of *Klebsiella pneumoniae* and *Pseudomonas aeruginosa*, bacteria both resistant to 19 different antibiotics. Anacardic acid and totarol have also been combined with methicillin and have demonstrated positive results in suppressing strains of *Staphylococcus aureus* resistant to methicillin [21].

This review collects the investigations carried out to evaluate the antimicrobial activity of edible plants and macroalgae extracts, particularly focusing on Rhodophyta (red algae) and Chlorophyta (green algae), as they are classified in kingdom Plantae; therefore, no brown algae are considered in the present review [22].

Macroalgae extracts present several biological activities; among them, antibacterial activity is widely exhibited in plants and macroalgae [23,24,25,26]. To live in harsh environmental conditions and to fight back predators, macroalgae naturally developed secondary metabolites with a wide diversity of structural and biological properties. Additionally, differences in the inhibitory action of macroalgae extracts against microorganisms largely depend on a number of variables, including habitat and location of collection, macroalga developmental stage, ecological characteristics (irradiance and nutrients), and seasonality [27]. The antibacterial activity of macroalgae inhibited the growth of a wide array of bacteria; therefore, the incorporation of these compounds could reduce the use of the synthetic compounds that have caused antimicrobial resistance. From several investigations, red algae present a higher diversity of secondary metabolites than brown and green algae. The chemistry of Rhodophyta is dominated mainly from halogenated compounds, which exhibited diverse biological activities including anti-bacterial, antifungal, anti-inflammation, cytotoxic, and insecticidal activity [28,29].

Macroalgal metabolites are significantly different from terrestrial plant extracts; indeed, this is inspiring for scientists to explore new classes of bioactive compounds that can be involved in antibiotic development and research.

Even though the antimicrobial effects of plants and algal extracts as well as isolated phytochemical compounds are undeniable, more clinical studies should be carried out to determine the optimal conditions under which these compounds could be safely consumed alone or in combination with conventional antibiotics.

The present review mainly collects literature regarding antimicrobial assays in vitro performed with extracts of terrestrial edible plants, and macroalgae, in particular Rhodophyta and Chlorophyta, belonging to the Plantae kingdom. The research methodology consists of research on Google Scholar and PubMed web search engines of recent literature using the keywords “antimicrobial assays of edible plants”; “red algae with antimicrobial activity”; “green algae with antimicrobial activity”; “antimicrobial resistance”; etc. From the articles of interest, the authors select the useful bibliography suitable for the review scope. The considered publications are between the years 2018 and 2023, as the authors wanted to focus on the most recent investigations. This review contains 144 references: 12 are related to plant extracts with antimicrobial activity (counting 25 plant extracts), 10 and 14 articles investigate Rhodophyta and Chlorophyta, respectively, counting 13 red and 17 green algae extracts exhibiting antibacterial properties.

## 2. Causes of Antimicrobial Resistance

Antimicrobial resistance is a major global concern for people and animals’ health. According to a report by the Organization for Economic Co-operation and Development (OECD), if there is no stop to antibiotic resistance, it may result in an increase in the number of deaths of 2.4 million people in Europe, Australia, and North America by 2050, with 90,000 or 1.3 million of deaths expected in Europe. According to the most recent assessments, antibiotic resistance is “one of the biggest risks to modern medicine”. Currently, sepsis brought by bacteria strains resistant to antibiotics results in 44,000 deaths every year in the UK [30].

Thomson [31] reports that Western sub-Saharan Africa had the highest rate of mortality directly attributed to AMR among the 21 GBD geographic regions, with 27.3 per 100,000 deaths. It is more likely to have highest death mortality rate due to antimicrobial resistance in low-income areas compared to wealthy nations, as both the prevalence of resistance and the number of infections with resistant bacteria are higher. Multidrug-resistant bacteria are accountable for antibiotic resistance, due to the development, dissemination, and persistence of the bacteria [32]. Antibiotics that are supplied over the counter which leads to their overuse, increased worldwide travel, inadequate sanitation/hygiene conditions, and the release of non-metabolized antibiotics or residues into the environment through manure/feces are a few of the possible reasons for antimicrobial resistance. These elements add to the genetic selection pressure that leads to the spread of bacterial illnesses that are resistant to a variety of treatments. Darwinian selection forced bacteria to develop a robust defense against the damaging impacts of antimicrobial agents. The majority of antibiotics are naturally manufactured by microbes such as saprophytic bacteria or fungi, while some, such as fluoroquinolones and sulphonamides, are entirely synthetic [33].

Antibiotic misuse is severely discouraged; however, there are still too many prescriptions written worldwide. According to several studies, from 30% to 50% of the cases, treatment indications, agent selection, and antibiotic medication length are ineffective; therefore, antimicrobial resistance is accentuated [34,35,36]. One of the causes of microbial resistance is the lack of monitoring of resistance development. The first report on antimicrobial resistance was published by the World Health Organization (WHO) in 2014, and it included data gathered from nine bacterial infections/antibiotic combinations that caused more health complications. Data showed that just 129 of the 194 studied countries had given information, and only 22 of those had information on all nine infection–antibiotic resistance combinations [37]. Surveillance is fundamental to monitor the status of antibiotics to be efficient against the interest bacterial strains. Mostly in many developing countries, poor quality of available antibiotics is the reason for having microbial resistance; tropical conditions and no possibility to store the antibiotics at the right temperature, in addition to a lack of proper transport methods, affect the quality of the antibiotics [38]. The easy availability of antibiotics is also reflected in their overuse, contributing to microbial resistance. Lastly, clinical misdiagnosis and antibiotic misuse also contribute to antibiotic resistance due to the excessive use of antibiotics when not clinically indicated [39].

**Figure 1:**
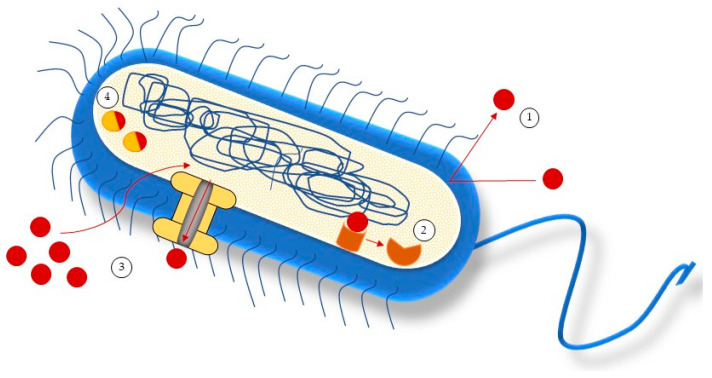
Mechanism of antimicrobial resistance. (1) Impermeability; (2) Modification; (3) Pumping out; (4) Inactivation.

The main mechanisms by which microorganisms exhibit resistance to antimicrobials [40] are shown in Figure 1. There is a change in the permeability of the bacterial cell wall (1) due to the modification of the cell wall proteins; therefore, the antibiotic cannot go beyond the cell wall. Next, there is target site alteration (2), which frequently results from a bacterial gene’s spontaneous mutation. Minor changes to the target molecule can have a significant impact on antibiotic action since antibiotic interactions with targets are typically rather specific (2). With an increased activity of the efflux pumps, the proteins present in the cytoplasmic membrane discharge the antibiotics outside the bacterial wall and maintain intracellular concentrations low (3) [41]. Moreover, the inactivation of the antibiotic through the bond with a phosphate group (4) reduces the ability of the antibiotic to bind with bacterial ribosomes [42]. Bacterial ribosomes are the major bacterial targets for antibiotics, since drugs inhibit ribosome function by interfering in messenger RNA translation or by blocking the formation of bacterial peptide bonds and proteins, inhibiting peptidyl transferase.

## 3. An Overview of Terrestrial Plants’ Antibacterial Activity

After the discovery of penicillin and the extension of its therapeutic use, other antibiotics were created for the daily treatment of infections, leading to an increase in the clinical usage of both natural and chemical antibiotics. However, most bacteria now have increased resistance to different antibiotics after the overuse of these treatments [43]. This is one of the reasons for the growing interest in researching natural antimicrobial compounds with low-risk in antimicrobial resistance, affordable, and comparable to synthetic antibiotics in the treatment of bacterial infections [44].

To overcome the crisis of antibiotic resistance, extracts from edible plants have been investigated to determine their antimicrobial activity, since natural products offer a promising source of antibacterial chemicals that could help to fill the drug discovery pipeline [45,46,47]. Due to the diverse and abundant plant biodiversity, there are numerous antibacterial compounds available in nature. Each plant evolved sophisticated defense mechanisms to fight bacteria, utilizing a variety of different and unique secondary compounds [48].

Currently, studies have been carried out on the use of edible plants to cure bacterial infections (Table 1). Gonelimali et al. [49] evaluated the antimicrobial potential of ethanolic and water extracts of aromatic plants widely used in cooking. *Thymus vulgaris*, known as thyme, *Rosmarinus officinalis* (rosemary), *Syzygium aromaticum* (clove), and *Hibiscus sabdariffa* (roselle) demonstrated antimicrobial activity against food pathogens and spoilage microorganisms, including *Bacillus cereus*, *S. aureus*, *Escherichia coli*, *Salmonella enteritidis*, *Vibrio parahaemolyticus*, and *P. aeruginosa*.

The water extracts for all four plants, except for *T. vulgaris*, exhibited the highest inhibition activity for *B. cereus*. The *T. vulgaris* water extract had a zone inhibition of 13.9 ± 1.3 mm against *V. parahaemolyticus*, while the ethanolic extract was the most effective against *B. cereus* with an inhibition zone of 17.3 ± 0.7 mm. The *R. officinalis* ethanolic extract displayed a high inhibition zone against *S. aureus* (19.8 ± 0.4 mm), while *B. cereus* and *P. aeruginosa* were highly inhibited from the ethanolic extract of *S. aromaticum* and *H. sabdariffa*, respectively, with inhibition zones of 18.2 ± 3.2 mm and 23.4 + 1.4 mm. The minimum inhibitory concentration (MIC) of these extracts has been reported in Table 1 [49].

The antimicrobial properties of thyme have also been proven by Burt et al. [50]; the authors considered that its antibacterial activity may be due to the hydrophobic bonding and hydrogen bonding between thymol, a phenol present in thyme which presents antibacterial activity, and the membrane proteins of cells, changing the permeability of the membranes and avoiding the entrance of the bacteria into the cells.

*Glycyrrhiza glabra* L., also known as liquorice, has been widely used for its medical properties. The first report of its medicinal use comes from Greeks, who recommended it for the treatment of gastric and peptic ulcers. In Asia and Europe, the extract is used in the treatment of psoriasis. It is used in traditional Chinese medicine to treat gastrointestinal disorders and oral ulcers [51], hepatitis, and heart disease [52,53]. Its extract is suitable as an adjuvant for inhibiting the growth of colon cancer cells such as prostate cancer [54] and gastric cancer [55]. Liquorice is a traditional medicinal herb that grows in different parts of the world. It is a very sweet, moist, soothing herb that detoxifies and protects the liver, and it also has powerful anti-inflammatory applications in arthritis and mouth ulcers.

The results of Gupta et al. [56] indicate the potential use of liquorice as antitubercular agent through systemic experiments. The antimycobacterial activity of the root ethanolic extract of *G. glabra* L. was observed at 500 µg/mL against *Mycobacterium tuberculosis* H37Ra and H37Rv strains.

According to the result of the research conducted by Jafari-Sales et al. [57], *S. aureus*, *B. cereus*, *E. coli,* and *P. aeruginosa* were affected from the methanolic extracts of *G. glabra* L. in both the agar well diffusion and dilution test techniques. *S. aureus* had the greatest response, whereas *P. aeruginosa* demonstrated the weakest response, as we see from the MIC values in Table 1. As a result, it is reasonable to assume that the *G. glabra* L. extract can be used to treat diseases caused by pathogenic bacteria and may be ideal for the formulation of natural antibiotics.

The antimicrobial effects of ethanolic extracts of five herbal plants, namely guava (*Psidium guajava*), sage (*Salvia officinalis*), rhamnus (*Ziziphusspina christi*), mulberry (*Morus alba*), and olive (*Olea europaea*) leaves were investigated to evaluate the growth inhibition of *S. aureus*, *E. coli*, *Pasteurella multocida*, *B. cereus,* and *S. enteritidis*. The results show that *Psidium guajava* inhibited *S. aureus* and *P. multocida* growth. *Salvia officinalis* exhibited a high growth inhibition for *S. aureus*, *E. coli*, and *S. enteritidis*. *O. europaea* resulted to be powerful against *B. cereus*, *E. coli,* and *S. enteritidis* growth. *M. alba* has been detected to have antibacterial potency against *B. cereus* and *P. multocida*. Therefore, this investigation confirms the idea to involve natural herbal extracts in antibiotic development [58].

The chemical composition and antimicrobial activities of nine wild edible Mediterranean species, namely *Reichardia picroides*, *Hymenonema graecum*, *Sonchus oleraceus*, *Scolymus hispanicus*, *Hedypnois cretica*, *Picris echioides*, *Urospermum picroides*, *Taraxacum* sp., and *Taraxacum officinale*, were investigated. The human pathogenic microorganisms that were tested include *Salmonella typhimurium*, *Listeria monocytogenes*, *E. coli*, *B. cereus*, and *S. aureus*. For each examined species, significant antibacterial activity was detected against *B. cereus* and *S. typhimurium*. With the exception of the *Taraxacum* species, flavonoids were the most prevalent phenolic compounds based on their chemical makeup in all the species.

Additionally, by commercial culture techniques and planning the growing season, the production of the investigated species may be enhanced and their commercial value could be boosted in the pharmaceutical and nutraceutical fields. Research on the antibacterial activities of plant extracts revealed encouraging findings that may be applied to the food processing sector as substitute food preservatives or food products with antimicrobic properties.

Therefore, these species could be viewed as useful components of nutritious diets that improve consumer welfare while also ensuring year-round availability [59].

The antibacterial properties of plant extracts are influenced by the extraction techniques. Five Thai edible plant leaf extracts, including *Anacardium occidentale* L., *Garcinia cowa*, *Glochidion wallichianum*, *Careya sphaerica*, and *Gnetum gnemon* var. *temerum*, were explored by Junsathian et al. [60] for their total phenolic content (TPC), total flavonoid content (TFC), antioxidant, and antimicrobial properties. The authors performed different extraction methods, such as ethanol extraction (EE), microwave-assisted extraction (MAE), and ultrasonic-assisted extraction (UAE). Compared to the UAE and EE techniques, the MAE leaf extracts of *G. wallichianum* had the highest extraction yield. The antioxidant and antibacterial activity of TPC and TFC were greater in the MAE-extracted *C. sphaerica* and *A. occidentale* L. samples. All extracts that had undergone MAE processing, with the exception of *A. murica* extracts, which may have required higher concentrations to show activity, had the greatest MIC and zones of inhibition against Gram-positive and Gram-negative bacterial strains. Consequently, MAE is a promising technique for obtaining bioactive substances. These results suggest that MAE enhanced the antioxidant and antibacterial efficiency of the leaf extracts in comparison to EE and UAE. The aforementioned extracts could be used as natural food additives to stop food from becoming spoiled by bacteria.

Therefore, the uses of common and edible plants in pharmaceutical field are numerous, as we can see in numerous published papers and literature reviews [61,62,63,64].

**Table 1:** Antimicrobial activity of edible terrestrial plants (“–“ = no antimicrobial activity revealed; “1GP” = 1st growing period; “2GP” = 2nd growing period).

Terrestrial Plant	Extract Type	Microbes	Minimum Inhibitory Concentration (MIC)	Inhibition Zone Diameter (mm)	Reference
*Anacardium occidentale*	Ethanolic extract	*Staphylococcus* *aureus*	1.56 mg/mL	16.00	[60]
		*Bacillus subtilis*	1.56 mg/mL	15.00	
		*Escherichia coli*	1.56 mg/mL	12.00	
		*Pseudomonas aeruginosa*	1.56 mg/mL	14.00	
	UAE	*Staphylococcus* *aureus*	<1.56 mg/mL	14.00	
		*Bacillus subtilis*	<1.56 mg/mL	16.00	
		*Escherichia coli*	1.56 mg/mL	14.00	
		*Pseudomonas aeruginosa*	1.56 mg/mL	15.00	
	MAE	*Staphylococcus* *aureus*	0.78 mg/mL	17.00	
		*Bacillus subtilis*	<1.56 mg/mL	16.00	
		*Escherichia coli*	1.56 mg/mL	15.00	
		*Pseudomonas aeruginosa*	1.56 mg/mL	16.00	
*Careya sphaerica*	Ethanolic extract	*Staphylococcus* *aureus*	<1.56 mg/mL	17.00	[60]
		*Bacillus subtilis*	<1.56 mg/mL	17.00	
		*Escherichia coli*	3.12 mg/mL	11.00	
		*Pseudomonas aeruginosa*	1.56 mg/mL	13.00	
	UAE	*Staphylococcus* *aureus*	<1.56 mg/mL	17.00	
		*Bacillus subtilis*	<1.56 mg/mL	16.00	
		*Escherichia coli*	3.12 mg/mL	15.00	
		*Pseudomonas aeruginosa*	1.56 mg/mL	14.00	
	MAE	*Staphylococcus* *aureus*	<1.56 mg/mL	18.00	
		*Bacillus subtilis*	<1.56 mg/mL	17.00	
		*Escherichia coli*	1.56 mg/mL	16.00	
		*Pseudomonas aeruginosa*	1.56 mg/mL	16.00	
*Garcinia cowa*	Ethanolic extract	*Staphylococcus* *aureus*	3.12 mg/mL	14.00	[60]
		*Bacillus subtilis*	1.56 mg/mL	11.00	
		*Escherichia coli*	12.5 mg/mL	11.00	
		*Pseudomonas aeruginosa*	12.5 mg/mL	12.00	
	UAE	*Staphylococcus* *aureus*	<1.56 mg/mL	14.00	
		*Bacillus subtilis*	<1.56 mg/mL	16.00	
		*Escherichia coli*	12.5 mg/mL	11.00	
		*Pseudomonas aeruginosa*	12.5 mg/mL	13.00	
	MAE	*Staphylococcus* *aureus*	0.78 mg/mL	14.00	
		*Bacillus subtilis*	<1.56 mg/mL	16.00	
		*Escherichia coli*	12.5 mg/mL	11.00	
		*Pseudomonas aeruginosa*	12.5 mg/mL	12.00	
*Glochidion wallichianum*	Ethanolic extract	*Staphylococcus* *aureus*	<1.56 mg/mL	16.00	[60]
		*Bacillus subtilis*	<1.56 mg/mL	14.00	
		*Escherichia coli*	6.25 mg/mL	10.00	
		*Pseudomonas aeruginosa*	3.12 mg/mL	13.00	
	UAE	*Staphylococcus* *aureus*	<1.56 mg/mL	14.00	
		*Bacillus subtilis*	<1.56 mg/mL	15.00	
		*Escherichia coli*	3.12 mg/mL	15.00	
		*Pseudomonas aeruginosa*	6.25 mg/mL	13.00	
	MAE	*Staphylococcus* *aureus*	0.78 mg/mL	14.00	
		*Bacillus subtilis*	<1.56 mg/mL	15.00	
		*Escherichia coli*	3.12 mg/mL	15.00	
		*Pseudomonas aeruginosa*	3.12 mg/mL	13.00	
*Glycyrrhiza glabra*	Ethanolic extract	*Mycobacterium* *tuberculosis H37Ra*	500 mg/mL	-	[56]
		*Mycobacterium* *tuberculosis H37Rv*	500 mg/mL	-	
*Glycyrrhiza glabra*	Methanolic extract	*Staphylococcus* *aureus*	6.25 mg/mL	10 ± 1.34	[57]
		*Bacillus* *cereus*	12.5 mg/mL	7 ± 1	
		*Escherichia* *coli*	50 mg/mL	6 ± 1.22	
		*Pseudomonas aeruginosa*	100 mg/mL	-	
*Gnetum gnemon* var. *temerum*	Ethanolic extract	*Staphylococcus* *aureus*	50.00 mg/mL	10.00	[60]
		*Bacillus subtilis*	25.00 mg/mL	-	
		*Escherichia coli*	50.00 mg/mL	9.00	
		*Pseudomonas aeruginosa*	50.00 mg/mL	9.00	
	UAE	*Staphylococcus* *aureus*	50 mg/mL	10.00	
		*Bacillus subtilis*	12.5 mg/mL	-	
		*Escherichia coli*	50 mg/mL	10.00	
		*Pseudomonas aeruginosa*	25 mg/mL	10.00	
	MAE	*Staphylococcus* *aureus*	12.5 mg/mL	11.00	
		*Bacillus subtilis*	6.25 mg/mL	-	
		*Escherichia coli*	50 mg/mL	11.00	
		*Pseudomonas aeruginosa*	25 mg/mL	10.00	
*Hedypnois cretica*	Methanolic extract	*Bacillus* *cereus*	2GP: 0.15 mg/mL	-	[59]
		*Staphylococcus* *aureus*	2GP: 0.60 mg/mL	-	
		*Listeria monocytogenes*	2GP: 0.45 mg/mL	-	
		*Escherichia* *coli*	2GP: 0.20 mg/mL	-	
		*Enterobacter* *cloacae*	2GP: 0.30 mg/mL	-	
		*Salmonella typhimurium*	2GP: 0.30 mg/mL	-	
*Hibiscus sabdariffa*	Ethanolic extract	*Bacillus cereus*	5 (% w/v)	22.2 + 0.8	[49]
	Water extract		0.625 (% w/v)	17.0 + 1.1	
	Ethanolic extract	*Staphylococcus aureus*	2.5 (% w/v)	21.5 + 2.1	
	Water extract		2.5 (% w/v)	15.7 + 1.0	
	Ethanolic extract	*Escherichia coli*	5 (% w/v)	21.1 + 1.3	
	Water extract		5 (% w/v)	15.6 + 1.2	
	Ethanolic extract	*Salmonella enteritidis*	5 (% w/v)	20.2 + 1.7	
	Water extract		10 (% w/v)	14.0 + 1.9	
	Ethanolic extract	*Vibrio parahaemolyticus*	2.5 (% w/v)	20.3 + 1.8	
	Water extract		5 (% w/v)	15.9 + 1.7	
	Ethanolic extract	*Pseudomonas aeruginosa*	2.5 (% w/v)	23.4 + 1.4	
	Water extract		5 (% w/v)	13.9 + 1.9	
*Hymenonema graecum*	Methanolic extract	*Bacillus* *cereus*	1GP: 0.20 mg/mL 2GP: 0.20 mg/mL	-	[59]
		*Staphylococcus* *aureus*	1GP: 0.60 mg/mL 2GP: 0.60 mg/mL	-	
		*Listeria monocytogenes*	1GP: 0.60 mg/mL 2GP: 0.60 mg/mL	-	
		*Escherichia* *coli*	1GP: 0.60 mg/mL 2GP: 0.60 mg/mL	-	
		*Enterobacter* *cloacae*	1GP: 0.45 mg/mL 2GP: 0.60 mg/mL	-	
		*Salmonella typhimurium*	1GP: 0.30 mg/mL 2GP: 0.30 mg/mL	-	
*Morus alba*	Ethanolic extract	*Staphylococcus* *aureus*	-	10.5 ± 1.15	[58]
		*Bacillus* *cereus*	2500 µg/mL	14.75 ± 0.15	
		*Escherichia* *coli*	-	7.5 ± 0.15	
		*Pasteurella* *multocida*	1250 µg/mL	15.42 ± 0.15	
		*Salmonella enteritidis*	625 µg/mL	12.02 ± 0.05	
*Olea europaea*	Ethanolic extract	*Staphylococcus* *aureus*	625 µg/mL	12.02 ± 2.05	[58]
		*Bacillus* *cereus*	5000 µg/mL	16.62 ± 1.05	
		*Escherichia* *coli*	2500 µg/mL	16.72 ± 0.55	
		*Pasteurella* *multocida*	625 µg/mL	9.12 ± 0.05	
		*Salmonella enteritidis*	5000 µg/mL	18.02 ± 0.05	
*Picris echioides*	Methanolic extract	*Bacillus* *cereus*	1GP: 0.075 mg/mL 2GP: 0.15 mg/mL	-	[59]
		*Staphylococcus* *aureus*	1GP: 0.45 mg/mL 2GP: 0.30 mg/mL	-	
		*Listeria monocytogenes*	1GP: 0.60 mg/mL 2GP: 0.30 mg/mL	-	
		*Escherichia* *coli*	1GP: 0.45 mg/mL 2GP: 0.15 mg/mL	-	
		*Enterobacter* *cloacae*	1GP: 0.30 mg/mL 2GP: 0.20 mg/mL	-	
		*Salmonella typhimurium*	1GP: 0.60 mg/mL 2GP: 0.20 mg/mL	-	
*Psidium guajava*	Ethanolic extract	*Staphylococcus* *aureus*	1250 µg/mL	15.62 ± 1.15	[58]
		*Bacillus* *cereus*	-	10.05 ± 0.15	
		*Escherichia* *coli*	625 µg/mL	10.55 ± 0.15	
		*Pasteurella* *multocida*	5000 µg/mL	18.02 ± 0.95	
		*Salmonella enteritidis*	625 µg/mL	10.12 ± 0.55	
*Reichardia picroides*	Methanolic extract	*Bacillus* *cereus*	1GP: 0.15 mg/mL 2GP: 0.15 mg/mL	-	[59]
		*Staphylococcus* *aureus*	1GP: 0.30 mg/mL 2GP: 0.30 mg/mL	-	
		*Listeria monocytogenes*	1GP: 0.30 mg/mL 2GP: 0.30 mg/mL	-	
		*Escherichia* *coli*	1GP: 0.15 mg/mL 2GP: 0.30 mg/mL	-	
		*Enterobacter* *cloacae*	1GP: 0.30 mg/mL 2GP: 0.30 mg/mL	-	
		*Salmonella typhimurium*	1GP: 0.30 mg/mL 2GP: 0.60 mg/mL	-	
*Rosmarinus officinalis*	Ethanolic extract	*Bacillus cereus*	5 (% w/v)	19.8 ± 0.8	[49]
	Water extract		1.25 (% w/v)	13.9 ± 1.2	
	Ethanolic extract	*Staphylococcus aureus*	1.25 (% w/v)	19.8 ± 0.4	
	Water extract		20 (% w/v)	12.7 ± 0.4	
	Ethanolic extract	*Escherichia coli*	5 (% w/v)	21.1 ± 0.9	
	Water extract		20 (% w/v)	12.5 ± 0.7	
	Ethanolic extract	*Salmonella enteritidis*	2.5 (% w/v)	20.7 ± 1.2	
	Ethanolic extract	*Vibrio parahaemolyticus*	-	-	
	Water extract		-	-	
	Ethanolic extract	*Pseudomonas aeruginosa*	-	-	
	Water extract		-	-	
*Salvia officinalis*	Ethanolic extract	*Staphylococcus* *aureus*	5000 µg/mL	17.05 ± 1.05	[58]
		*Bacillus* *cereus*	625 µg/mL	16.45 ± 1.05	
		*Escherichia* *coli*	2500 µg/mL	19.25 ± 0.65	
		*Pasteurella* *multocida*	-	9.05 ± 1.05	
		*Salmonella enteritidis*	2500 µg/mL	16.25 ± 0.75	
*Scolymus hispanicus*	Methanolic extract	*Bacillus* *cereus*	2GP: 0.10 mg/mL	-	[59]
		*Staphylococcus* *aureus*	2GP: 0.30 mg/mL	-	
		*Listeria monocytogenes*	2GP: 0.20 mg/mL	-	
		*Escherichia* *coli*	2GP: 0.10 mg/mL	-	
		*Enterobacter* *cloacae*	2GP: 0.15 mg/mL	-	
		*Salmonella typhimurium*	2GP: 0.15 mg/mL	-	
*Sonchus oleraceus*	Methanolic extract	*Bacillus* *cereus*	1GP: 0.20 mg/mL 2GP: 0.15 mg/mL	-	[59]
		*Staphylococcus* *aureus*	1GP: 0.45 mg/mL 2GP: 0.30 mg/mL	-	
		*Listeria monocytogenes*	1GP: 0.45 mg/mL 2GP: 0.60 mg/mL	-	
		*Escherichia* *coli*	1GP: 0.45 mg/mL 2GP: 0.30 mg/mL	-	
		*Enterobacter* *cloacae*	1GP: 0.60 mg/mL 2GP: 0.30 mg/mL	-	
		*Salmonella typhimurium*	1GP: 0.45 mg/mL 2GP: 0.30 mg/mL	-	
*Syzygium aromaticum*	Ethanolic extract	*Bacillus cereus*	2.5 (% w/v)	18.2 ± 3.2	[49]
	Water extract		0.313 (% w/v)	15.1 ± 0.9	
	Ethanolic extract	*Staphylococcus aureus*	2.5 (% w/v)	16.7 ± 1.0	
	Water extract		5 (% w/v)	13.6 ± 1.3	
	Ethanolic extract	*Escherichia coli*	2.5 (% w/v)	17.4 ± 0.8	
	Water extract		5 (% w/v)	13.2 ± 1.6	
	Ethanolic extract	*Salmonella enteritidis*	5 (% w/v)	15.1 ± 1.4	
	Water extract		5 (% w/v)	12.2 ± 1.1	
	Ethanolic extract	*Vibrio parahaemolyticus*	0.625 (% w/v)	14.7 ± 2.0	
	Water extract		2.5 (% w/v)	13.1 ± 1.8	
	Ethanolic extract	*Pseudomonas aeruginosa*	5 (% w/v)	17.0 ± 0.5	
	Water extract		10 (% w/v)	13.2 ± 1.4	
*Taraxacum officinale*	Methanolic extract	*Bacillus* *cereus*	1GP: 0.037 mg/mL 2GP: 0.20 mg/mL	-	[59]
		*Staphylococcus aureus*	1GP: 0.30 mg/mL 2GP: 0.90 mg/mL	-	
		*Listeria monocytogenes*	1GP: 0.30 mg/mL 2GP: 0.90 mg/mL	-	
		*Escherichia* *coli*	1GP: 0.15 mg/mL 2GP: 0.30 mg/mL	-	
		*Enterobacter* *cloacae*	1GP: 0.15 mg/mL 2GP: 0.30 mg/mL	-	
		*Salmonella typhimurium*	1GP: 0.15 mg/mL 2GP: 0.60 mg/mL	-	
*Taraxacum* sp.	Methanolic extract	*Bacillus* *cereus*	1GP: 0.075 mg/mL 2GP: 0.075 mg/mL	-	[59]
		*Staphylococcus* *aureus*	1GP: 0.60 mg/mL 2GP: 0.30 mg/mL	-	
		*Enterobacter* *monocytogenes*	1GP: 0.45 mg/mL 2GP: 0.45 mg/mL	-	
		*Escherichia* *coli*	1GP: 0.20 mg/mL 2GP: 0.90 mg/mL	-	
		*Enterobacter* *cloacae*	1GP: 0.20 mg/mL 2GP: 0.20 mg/mL	-	
		*Salmonella typhimurium*	1GP: 0.20 mg/mL 2GP: 0.30 mg/mL	-	
*Thymus vulgaris*	Ethanolic extract	*Bacillus cereus*	5 (% w/v)	17.3 ± 0.7	[49]
	Water extract		5 (% w/v)	13.8 ± 1.1	
	Ethanolic extract	*Staphylococcus aureus*	5 (% w/v)	15.9 ± 0.3	
	Water extract		2.5 (% w/v)	12.2 ± 0.7	
	Ethanolic extract	*Escherichia coli*	10 (% w/v)	15.9 ± 0.3	
	Water extract		5 (% w/v)	12.2 ± 0.7	
	Water extract	*Salmonella enteritidis*	5 (% w/v)	11.8 ± 1.4	
	Water extract	*Vibrio parahaemolyticus*	2.5 (% w/v)	13.9 ± 1.3	
	Ethanolic extract		10 (% w/v)	14.3 ± 0.1	
	Water extract	*Pseudomonas aeruginosa*	-	-	
	Ethanolic extract		-	-	
*Urospermum picroides*	Methanolic extract	*Bacillus* *cereus*	1GP: 0.15 mg/mL 2GP: 0.15 mg/mL	-	[59]
		*Staphylococcus* *aureus*	1GP: 0.90 mg/mL 2GP: 0.90 mg/mL	-	
		*Listeria monocytogenes*	1GP: 0.90 mg/mL 2GP: 0.30 mg/mL	-	
		*Escherichia* *coli*	1GP: 0. 90 mg/mL 2GP: 0.45 mg/mL	-	
		*Enterobacter cloacae*	1GP: 0.45 mg/mL 2GP: 0.60 mg/mL	-	
		*Salmonella typhimurium*	1GP: 0.45 mg/mL 2GP: 0.30 mg/mL	-	
*Ziziphusspina christi*	Ethanolic extract	*Staphylococcus* *aureus*	625 µg/mL	11.82 ± 2.5	[58]
		*Bacillus* *cereus*	625 µg/mL	13.52 ± 2.1	
		*Escherichia* *coli*	-	10.02 ± 0.05	
		*Pasteurella* *multocida*	-	8.52 ± 2.5	
		*Salmonella enteritidis*	625 µg/mL	12.82 ± 2.5	

Not only terrestrial plants possess bioactive compounds with interesting properties. In recent decades, research on marine plants and algae as well as biological compounds has been carried out, with impressive outcomes that demonstrate the potential therapeutic activity of compounds of marine origin.

## 4. Antimicrobial Activity from Rhodophyta and Chlorophyta Extracts

Macroalgae are aquatic photosynthetic organisms (mainly marine) belonging to the domain Eukarya. Macroalgae are mainly divided into three groups: red algae (Rhodophyta) and green algae (Chlorophyta), which are classified in kingdom Plantae, and brown algae (Ochrophyta, class Phaeophyceae), belonging to kingdom Chromista [22]. Therefore, as terrestrial plants, macroalgae possess interesting biological activities that could be involved in the development of natural and innovative antibiotics [65]. Macroalgae’s biological activities can vary among phyla [66]. In the following section, antimicrobial tests for Rhodophyta and Chlorophyta extracts are considered.

### 4.1. Rhodophyta

Two different extracts of *Gracilaria corticata* and *Gracilaria edulis* (methanolic and dimethyl sulfoxide (DMSO) extracts), were investigated against pathogenic bacteria such as *E. coli*, *Bacillus subtilis*, *B. cereus*, *S. aureus*, *Photobacterium* sp., and *Pseudomonas fluorescens* [67]. All tested extracts exhibited antimicrobial activity against these pathogenic bacteria (Table 2), and GC-MS analysis has revealed the presence of numerous bioactive metabolites such as sulphurous acid, 2-ethylhexyl isohexyl ester, eugenol, benzene, and phthalic acid in both red macroalgae. Jasna et al. [68] reported high concentrations of eugenol in the clove extract, which proves its potential for antibacterial and antioxidant properties. The antibacterial mechanism of action of eugenol consists of the disruption of the cell structure by the incorporation within the lipopolysaccharides layer of the bacteria’s cell membrane, which leads to the intracellular components’ release and the death of the bacteria [69]. It may be possible that the same mechanism of actions happened with the *G. corticata* and *G. edulis* extracts.

**Figure 2:**
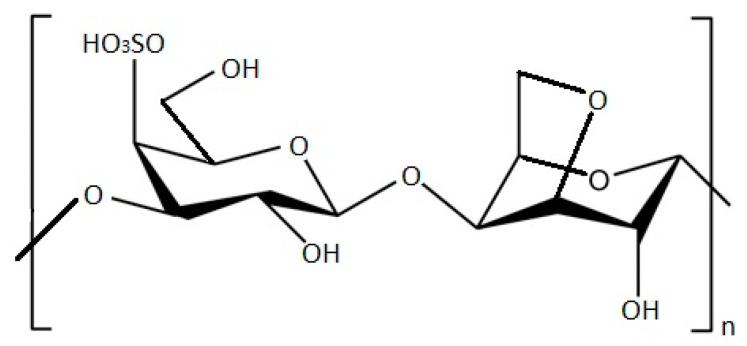
Structure of k-carrageenan molecule.

*S. aureus* and *E. coli* growth reductions have been shown by testing these pathogenic bacteria against *Grateloupia turuturu* ethanolic and polysaccharide extracts. The results show that both extracts revealed antibacterial activity, with polysaccharides exhibiting higher antimicrobial activity. The FTIR-ATR analysis made it possible to characterize *G. turuturu* polysaccharides, concluding that they are composed by a hybrid kappa/iota carrageenan (Figure 2) with traces of agar, in both phases of the life cycle. This suggests that these compounds may be responsible for this activity; therefore, this red alga may be of pharmaceutical interest, since it was possible to observe, both in the ethanolic extracts and polysaccharides extracts, the ability to inhibit the growth of two different bacterial strains [70].

Methanolic extracts of *G. edulis* and *Hypnea valentiae* were tested against human bacterial pathogens *Klebsiella oxytoca*, *E. coli*, *S. aureus*, *P. aeruginosa*, *B. subtilis*, *Serratia* sp., and *Salmonella* sp. The *G. edulis* polyphenol compound displayed a maximum of 23 mm of inhibition zone against *B. subtilis* and the *H. valentiae* polyphenol compound displayed a maximum of 17 mm of inhibition zone against *K. oxytoca* (Table 2). Polyphenols from red algae carry potential assets, as they may have a strong pharmaceutical value in the future. Biochemical analysis revealed the presence of flavonoids, saponins, tannin, and steroids in both red algae, while only *G. edulis* revealed phenolics and alkaloids [71].

The objective of the study of Freitas et al. [72] is to evaluate the antioxidant and antimicrobial activity of twelve red seaweed species commonly found on Portuguese shores, namely *Porphyra umbilicalis*, *Ceramium ciliatum*, *Osmundea pinnatifida, Chondrus crispus*, *Sphaerococcus coronopifolius*, *Plocamium cartilagineum*, *Corallina officinalis*, *Ellisolandia elongata*, *Amphiroa rigida*, *Jania rubens*, *Mesophyllum lichenoides,* and *Liagora viscida*. All of them possess interesting antimicrobial properties, and in Table 2 are reported the MIC and inhibition zone diameter of the edible seaweeds *P. umbilicalis*, *O. pinnatifida*, and *C. crispus*.

*P. umbilicalis* presents, by far, the highest phenol content when compared to the other studied algae, as well as a high scavenging ability. These results likely indicate strong antioxidant activity, as it is known that seaweeds are able to develop antioxidant shielding mechanisms and strategies to withstand highly oxidative environments [72].

The study of Bhuyar et al. [73] demonstrates that different extracts (water and ethanol) of red alga *K. alvarezii* were more efficient against *B. cereus* but not against *E. coli*, as disc diffusion assay results indicated.

Among edible seaweeds, *Pyropia orbicularis* [74] and *Asparagopsis taxiformis* both inhibited *S. aureus* and *E. coli*, with *Klebseilla* sp., *K. pneumoniae, Pseudomonas fluorescens, Vibrio proteolyticus,* and *Streptococcus* sp. *Bacillus subtilis* demonstrating a high inhibition zone [75] (Table 2).

The in vitro activity of the *Gelidium* sp. flour extract was evaluated against the most common pathogenic and spoilage bacteria. From the results, it emerged that *P. fluorescens* and *Pseudomonas putida* exhibited resistance to components of algal flour extract. Only *B. subtilis* and *Salmonella enterica* were inhibited by the lowest MIC. The highest level of inhibition was observed both for Gram-negatives such as Enterobacteriaceae (*E. coli*, *Enterobacter aerogenes*, and *K. pneumoniae*) and proteobacteria (*Vibrio alginolyticus*).

Due to the simplicity of the extraction methodology and the abundancy of *Gelidium* sp., further research is envisaged to optimize the extraction of the used compounds and to analyze the molecules involved in antimicrobial action [76].

Red algae are the main producers of halogenated compounds, which exhibited diverse biological activities including antibacterial, antifungal, anti-inflammatory, insecticidal, and carcinogenic effects. Along with several interesting amino acid, acetate, and nucleic acid derivatives, red algae also synthesize terpenoid, polyether, and acetogenin compounds [28,29]. For example, the halogenated sesquiterpene alcohol, elatol, is commonly found in *Laurencia* sp., and known for its potent antibacterial activity. The compound was isolated for the first time in *Laurencia microcladia*, collected in the Southern Brazilian coast, and tests showed the antiherbivore and antimicrobial activity of elatol [77].

**Table 2:** Antimicrobial activity of Rhodophyta species (“nd” = not determined; “–“ = no antimicrobial activity revealed).

Rhodophyta	Extract Type	Microbes	Minimum Inhibitory Concentration (MIC)	Inhibition Zone Diameter (mm)	Reference
*Asparagopsis* *taxiformis*	Methanolic extract	*Staphylococcus aureus*	0.5 mg/mL	>15	[75]
		*Serratia* sp.	0.5 mg/mL	-	
		*Klebseilla* sp.	0.5 mg/mL	>1	
		*Salmonella* sp.	0.5 mg/mL	-	
		*Escherichia coli*	0.5 mg/mL	>10	
		*Klebseilla pneumonia*	0.5 mg/mL	>1	
		*Pseudomonas aeruginosa*	0.5 mg/mL	-	
		*Pseudomonas fluorescens*	0.5 mg/mL	>10	
		*Vibrio proteolyticus*	0.5 mg/mL	>1	
		*Streptococcus* sp.	0.5 mg/mL	10	
		*Bacillus* *subtilis*	0.5 mg/mL	10	
*Chondrus crispus*		*Bacillus subtilis*	12.5 mg/mL	-	[72]
*Gelidium* sp.	Water extract	*Salmonella enterica*	12.5 mg/mL	>10	[76]
		*Klebsiella pneumoniae*	50 mg/mL	>11	
		*Listeria monocytogenes*	50 mg/mL	11	
		*Enterobacter aerogenes*	25 mg/mL	>11	
		*Proteus mirabilis*	50 mg/mL	>11	
		*Vibrio* *parahaemolyticus*	nd	>11	
		*Vibrio alginolyticus*	nd	13	
		*Bacillus licheniformis*	25 mg/mL	11	
		*Bacillus cereus*	0.625 mg/mL	>11	
		*Bacillus subtilis*	3.125 mg/mL	>10	
		*Escherichia coli*	50 mg/mL	>13	
		*Pseudomonas putida*	-	-	
		*Pseudomonas fluorescens*	-	-	
*Gracilaria corticata*	Methanolic extract	*Escherichia coli*	100 µg/mL	7 ± 0.01	[67]
		*Photobacterium* sp.	100 µg/mL	6 ± 0.04	
		*Pseudomonas fluorescens*	100 µg/mL	8 ± 0.1	
		*Staphylococcus aureus*	100 µg/mL	4 ± 0.10	
		*Bacillus subtilis*	100 µg/mL	8 ± 0.01	
	Dimethyl sulfoxide (DMSO) extract	*Escherichia coli*	100 µg/mL	5 ± 0.10	
		*Photobacterium* sp.	100 µg/mL	4 ± 0.30	
		*Pseudomonas fluorescens*	100 µg/mL	4 ± 0.05	
		*Staphylococcus aureus*	100 µg/mL	6 ± 0.05	
		*Bacillus subtilis*	100 µg/mL	5 ± 0.12	
*Gracilaria edulis*	Methanolic extract	*Escherichia coli*	100 µg/mL	3 ± 0.01	[67]
		*Photobacterium* sp.	100 µg/mL	1 ± 0.00	
		*Pseudomonas fluorescens*	100 µg/mL	3 ± 0.05	
		*Staphylococcus aureus*	100 µg/mL	3 ± 0.05	
		*Bacillus subtilis*	100 µg/mL	3 ± 0.03	
	Dimethyl sulfoxide (DMSO) extract	*Escherichia coli*	100 µg/mL	4.5 ± 0.01	
		*Photobacterium* sp.	100 µg/mL	4 ± 0.01	
		*Pseudomonas fluorescens*	100 µg/mL	4 ± 0.10	
		*Staphylococcus aureus*	100 µg/mL	3 ± 0.00	
*Gracilaria edulis*	Methanolic extracts	*Klebsiella oxytoca*	0.3 mg/mL	21	[71]
		*Escherichia coli*	0.3 mg/mL	19	
		*Staphylococcus aureus*	0.3 mg/mL	18	
		*Pseudomonas aeruginosa*	0.3 mg/mL	16	
		*Bacillus subtilis*	0.3 mg/mL	23	
		*Serratia* sp.	0.3 mg/mL	20	
		*Salmonella* sp.	0.3 mg/mL	22	
*Grateloupia turuturu*	Ethanolic extract	*Staphylococcus aureus*	10 mg/mL	-	[70]
		*Escherichia coli*	10 mg/mL	-	
	Polysaccharides (carrageenan)	*Staphylococcus aureus*	7.5 mg/mL	-	
		*Escherichia coli*	7.5 mg/mL	-	
*Hypnea valentiae*	Methanolic extract	*Klebsiella oxytoca*	0.3 mg/mL	17	[71]
		*Escherichia coli*	0.3 mg/mL	12	
		*Staphylococcus aureus*	0.3 mg/mL	14	
		*Pseudomonas aeruginosa*	0.3 mg/mL	11	
		*Bacillus subtilis*	0.3 mg/mL	15	
		*Serratia* sp.	0.3 mg/mL	13	
		*Salmonella* sp.	0.3 mg/mL	16	
*Kappaphycus alvarezii*	Ethanolic extract	*Escherichia coli*	-	-	[73]
		*Bacillus cereus*	0.5 mg/mL	<10	
	Hot water extract	*Escherichia coli*	-	-	
		*Bacillus cereus*	0.5 mg/mL	<10	
*Osmundea pinnatifida*		*Bacillus subtilis*	1.56 mg/mL	-	[72]
*Porphyra umbilicalis*	Aqueous extract	*Bacillus subtilis*	3.13 mg/mL	-	[72]
*Pyropia* *orbicularis*	Methanolic extract	*Staphylococcus aureus*	250 mg/mL	nd	[74]
		*Escherichia coli*	500 mg/mL		

### 4.2. Chlorophyta

*Caulerpa racemosa* and *Caulerpa lentillifera*, also known as “sea grapes”, are green seaweeds commonly found in different parts of the world. They are widely used as whole food, but they also possess interesting therapeutic properties. It has been investigated whether *C. racemosa* and *C. lentillifera* from Malaysia have antibacterial properties. Crude extracts from seaweed were obtained using chloroform, methanol, and water. The authors measured the total phenolic and flavonoid contents. Both seaweed extracts displayed antibacterial abilities against neuropathogenic *E. coli* K1 and methicillin-resistant *S. aureus* (MRSA). The results show that the *C. racemosa* chloroform extract had the highest total phenolic content and the strongest antibacterial effect against MRSA, but it did not demonstrate similar promising results against *E. coli* K1. The chloroform extract of *C. lentillifera* gives a moderate antibacterial effect on MRSA but poorly on *E. coli* K1. In both species, the methanol extracts only show a moderate antibacterial effect against both MRSA and *E. coli* K1. A positive correlation has been revealed between the TPC and antibacterial activity, suggesting that the antimicrobial action may be due to the presence of phenolics. Both the *C. racemosa* and *C. lentillifera* water extracts promote the growth of the bacteria. According to the study, *C. racemosa* chloroform extracts mostly contain polyunsaturated and monounsaturated fatty acids, terpenes, and alkaloids. As a result, *C. racemosa* has the potential to be an excellent source of new antibacterial compounds. Still, the mechanisms of action of these compounds are unclear; therefore, further studies are needed along with the improvement of isolation and purification techniques of bioactive compounds [78].

*C. racemosa*, along with *Ulva intestinalis*, have also been investigated to test their antibacterial activity against *Vibrio fluvialis*. It appears that both exhibit antibacterial activity against *V. fluvialis* bacteria, with *C. racemosa* exhibiting a higher activity [79].

Nagappan and Vairappan [80] evaluated the antibacterial properties of the green seaweed *C. lentillifera* and *C. racemosa* methanolic extracts against *E. coli*, *S. aureus*, *Streptococcus* sp., *Salmonella* sp., and *S. aureus*. The higher MIC has been determined for *C. racemosa* species against *Streptococcus* sp.

Ravikumar et al. [81] investigated *Caulerpa cuppressoides*, *Enteromorpha intestinalis,* and *Ulva lactuca* antimicrobial activity. Tests were carried out considering different extraction solvents, such as benzene, butanol, propanol, acetone, and water. The results reveal high inhibitory activities against *S. aureus, P. aeruginosa* for *C. cupressoides* propanol extracts, while for *Streptococcus pyogens* and *E. coli* with acetone extracts. *E. intestinalis* sees the highest antimicrobial activity for *K. pneumoniae* from water extracts, while *U. lactuca* exhibited a higher antimicrobial activity for butanol extracts against *S. aureus* and acetone extracts tested for *P. aeruginosa*.

*Ulva fasciata*, *U. lactuca*, *Cladophora vagabunda*, *Caulerpa taxifolia*, *Chaetomorpha anteninna*, and *Chaetomorpha linum* crude extracts were tested against *E. coli* clinical and laboratory strains, namely *E. coli* NCTC 10418, *E. coli* ATCC 25923, *Proteus vulgaris*, *P. mirabilis*, *P. aeruginosa*, *P. putida*, *Salmonella typhi* clinical strain, *Salmonella typhi* NCTC 8385, *Serratia macerans*, as well as *K. pneumoniae*, *S. aureus* ATCC 25922, *B. subtilis*, *Streptococcus pneumoniae*, *Enterobacter faecalis*, and *Mycobacterium aurum*.

The highest activity against the bacterial strain was detected in the diethyl acetate extract of *C. antennina* against *S. aureus* laboratory strain, while the highest inhibitory zone against the Gram-negative bacterial species was observed in the dichloromethane/methanol extract of *C. taxifolia* against *E. coli* strains. This alga presents the highest inhibitory activities among all the tested algae present in Table 3 that shows MIC and minimum zone of inhibition performed with the disc diffusion method. *U. fasciata* exhibited broad-spectrum antibacterial activity [82]. The effects of the methanol extracts of *Ulva* sp. against the following multidrug-resistant bacteria isolated from patients in Saudi Arabia and Malaysia were tested. The lowest MIC was for the 0.5 µg/mL extract of *Ulva* sp. against *S. agalactiae* (group B), whereas *S. saprophyticus* exhibited more resistance to *Ulva* sp. with an MIC of 16 µg/mL. This investigation conducted by Al-Zahrani et al. [83] is an example of the potential for obtaining new sources of antimicrobial agents to develop new therapeutically interesting molecules from easy cultivable seaweeds.

The results of Srikonga et al. [84], which evaluated the effects of the green seaweed *U. intestinalis* methanolic, ethanolic, dichloromethane, and hexane extracts, demonstrated antimicrobial activity against Gram-positive bacteria. The methanolic extracts exhibited activity against *B. cereus*, MRSA, and *S. aureus*, while the ethanolic and dichloromethane extracts affected only *L. monocytogenes*. All these four microbes are affected by the hexane extract. For these species, the authors calculated the MIC, shown in Table 3. The extract of *Enteromorpha* sp. had been tested by Swathi et al. [85] against *P. aeruginosa*, *S. aureus*, and *E. coli* to analyze its antibacterial activity by the disc diffusion method. At the concentrations of 150 g/mL and 200 g/mL, it produced a zone of clearance with diameters of 11 ± 0.2 mm and 13 ± 0.2 mm, respectively, against *P. aeruginosa*. It exhibited inhibitory zones of 10 ± 0.2 mm, 16 ± 0.2 mm, and 18 ± 0.2 mm at the concentrations of 100 g/mL, 150 g/mL, and 200 g/mL, respectively, for *S. aureus* and 11 ± 0.2 mm, 15 ± 0.2 mm, and 18 ± 0.2 mm, respectively, against *E. coli*. This demonstrated the excellent antioxidant and antibacterial properties of *Enteromorpha* sp. The bioactive compounds present in the green seaweed extract of *Enteromorpha compressa* were tested for its antimicrobial activity against human pathogens such as *Klebsiella* sp., *Salmonella* sp., *S. aureus,* and *Proteus* sp. *Salmonella* sp. was found to be more susceptible to *E. compressa* ethanolic extracts compared with the effect against other tested bacteria [86]. Phytochemical analysis confirmed the presence of phenols, alkaloids, flavonoids, steroids, and terpenoids that may be responsible for the antibacterial activity.

Cadar et al. [87] investigated the extracts of *U. lactuca* to determine total polyphenols content and antibacterial activity against *S. aureus, Staphylococcus epidermidis, P. aeruginosa,* and *E. coli*. Ampicillin was used as a standard drug and control. The chloroform extract demonstrated the largest inhibitory zone against *S. aureus*, comparable to that of conventional ampicillin. The extract in n-hexane displayed the biggest inhibitory zone against *Staphylococcus epidermides* and *P. aeruginosa*, comparable to the ampicillin control. The extracts in n-hexane and chloroform produced the largest areas of inhibition in the case of *E. coli*; however, they present low values compared to the ampicillin standard. The authors deduced from the tests that ampicillin-like antibacterial activity was present in n-hexane and chloroform extracts. Due to the presence of known bioactive chemical components that promote this property, *U. lactuca* validates its potential for antimicrobial properties [87].

The *Codium* species have received the least attention from exploring the biological activities of Chlorophyceae members for potential biomedical applications. *C. intricatum* methanol extract was tested for antibacterial activity against a variety of bacterial infections. It displayed a broad spectrum of inhibitory effects against MRSA and modest action against *B. cereus* and *L. monocytogenes* in a research conducted by Arguelles et al. [88].

However, in other studies, such as Koz et al.’s [89] investigation, the antibacterial activity of hexane, methanol, and dichloromethane of *Codium fragile* extracts against several pathogenic bacteria were tested. All three extracts of *C. fragile* demonstrated a similar weak antimicrobial activity on *B. subtilis*, MRSA, *E. aerogenes*, and *E. coli* compared with the standard antibiotic tobramycin.

The antibacterial activity of methanolic extracts of *C. bursa*, *C. tomentosum*, *C. dichotomum*, and *C. fragile* were tested against *S. aureus*, *E. coli*, *K. pneumoniae,* and *E. faecalis*. All *Codium* extracts exhibited high inhibition against *S. aureus*, except for *C. bursa*, for which no antibacterial activity was observed [90]. However, antimicrobial activity studies of *Codium* sp. are limited and need to be further developed.

**Figure 3:**
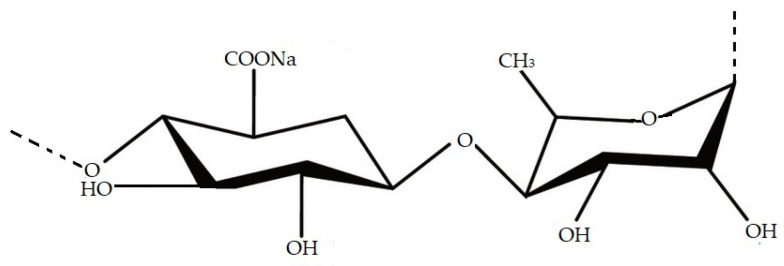
Structure of ulvan molecule.

Ulvan is a water-soluble sulphated polysaccharide (Figure 3) derived from marine green seaweed, which exhibits a wide range of physiological and biological activities such as anticancer [91], anticoagulant [92], antioxidant, antifungal, and antitumor activities [93,94]. Ulvan essentially contains rhamnose, xylose, glucuronic acid, iduronic acid, and sulphate groups [93,95] and its structure and properties can vary depending on algae species, place of cultivation, and method of extraction [96,97,98,99,100]. The study of Van Tran et al. [101] showed the antibacterial activity of ulvan extracted from *U. reticulata* against *E. coli*, *P. aeruginosa, and Enterobacter cloacae.* The highest inhibition activity was shown in *E. cloacae*, followed by *E. coli,* and the lowest inhibitor activity was in *P. aeruginosa* [101].

However, ulvan extracted from *U. lactuca* demonstrates activity against other pathogenic bacteria listed in Table 3 [102]. It is likely that the different antibacterial activities are due to molecular weight, density of charged groups, and morphology of molecules. Therefore, it is necessary to investigate more deeply the factors influencing the antibacterial activity of ulvan and its mechanisms of action.

**Table 3:** Antimicrobial activity of Chlorophyta species (“nd” = not determined; “–“ = no antimicrobial activity revealed).

Chlorophyta	Extract Type	Microbes	Minimum Inhibitory Concentration (MIC)	Inhibition Zone Diameter (mm)	Reference
*Caulerpa cupressoides*	Benzene	*Escherichia coli*	nd	6	[81]
		*Klebsiella pneumoniae*	nd	6	
		*Pseudomonas aeruginosa*	nd	5	
		*Streptococcus pyogens*	nd	6	
		*Staphylococcus aureus*	nd	6	
	Butanol	*Escherichia coli*	nd	7	
		*Klebsiella pneumoniae*	nd	-	
		*Pseudomonas aeruginosa*	nd	7	
		*Streptococcus pyogens*	nd	-	
		*Staphylococcus aureus*	nd	6	
	Propanol	*Escherichia coli*	nd	7	
		*Klebsiella pneumoniae*	nd	-	
		*Pseudomonas aeruginosa*	nd	8	
		*Streptococcus pyogens*	nd	7	
		*Staphylococcus aureus*	nd	6	
	Acetone	*Escherichia coli*	nd	9	
		*Klebsiella pneumoniae*	nd	6	
		*Pseudomonas aeruginosa*	nd	5	
		*Streptococcus pyogens*	nd	8	
		*Staphylococcus aureus*	nd	7	
	Water	*Escherichia coli*	nd	6	
		*Klebsiella pneumoniae*	nd	6	
		*Pseudomonas aeruginosa*	nd	-	
		*Streptococcus pyogens*	nd	-	
		*Staphylococcus aureus*	nd	-	
*Caulerpa lentillifera*	Water extract	Methicillin-resistant *Staphylococcus aureus*, *Escherichia coli*	5 µg/mL	nd	[78]
*Caulerpa lentillifera*	Methanolic extract	*Escherichia coli*	136.50 ± 0.85 mg/mL	nd	[80]
		*Staphylococcus aureus*	125.25 ± 3.78 mg/mL	nd	
		*Streptococcus* sp.	175.25 ± 0.23 mg/mL	nd	
		*Salmonella* sp.	140.50 ± 0.55 mg/mL	nd	
*Caulerpa racemosa*	Water extract	Methicillin-resistant *Staphylococcus aureus*, *Escherichia coli*	5 µg/mL	nd	[78]
*Caulerpa racemosa*	Methanolic extract	*Vibrio fluvialis*	nd	9 ± 0.50	[79]
*Caulerpa racemosa* var. *clavifera f. microphysa*	Methanolic extract	*Escherichia coli*	245.25 ± 2.11 mg/mL	nd	[80]
		*Staphylococcus aureus*	225.50 ± 0.45 mg/mL	nd	
		*Streptococcus* sp.	450.75 ± 1.09 mg/mL	nd	
		*Salmonella* sp.	275. 20 ± 0.66 mg/mL	nd	
*Caulerpa racemosa* var. *laetevirens*	Methanolic extract	*Escherichia coli*	360.50 ± 2.14 mg/mL	nd	[80]
		*Staphylococcus aureus*	375.75 ± 0.07 mg/mL	nd	
		*Streptococcus* sp.	450. 25 ± 0.42 mg/mL	nd	
		*Salmonella* sp.	345. 25 ± 0.35 mg/mL	nd	
*Caulerpa taxifolia*	Chloroform/methanol extract	*Escherichia coli*	640 µg/mL	7.33–10.67	[82]
		*Staphylococcus aureus*	-	10.00–11.17	
*Chaetomorpha anteninna*	Chloroform/methanol extract	*Escherichia coli*	640 µg/mL	7.33–10.67	[82]
		*Staphylococcus aureus*	640 µg/mL	10.00–11.17	
*Chaetomorpha linum*	Chloroform/methanol extract	*Escherichia coli*	>640 µg/mL	7.33–10.67	[82]
		*Staphylococcus aureus*	-	10.00–11.17	
*Cladophora vagabunda*	Chloroform/methanol extract	*Escherichia coli*	640 µg/mL	7.33–10.67	[82]
		*Staphylococcus aureus*	nd	10.00–11.17	
*Codium dichotomum*	Methanolic extract	*Staphylococcus aureus*	nd	≥20	[90]
		*Escherichia coli*	nd	<10	
		*Klebliella pneumoniae*	nd	<10	
		*Enterobacter faecalis*	nd	<10	
*Codium fragile*	Methanolic extract	*Staphylococcus aureus*	nd	≥20	[90]
		*Escherichia coli*	nd	<10	
		*Klebliella pneumoniae*	nd	<10	
		*Enterobacter faecalis*	nd	<10	
*Codium fragile*	Hexane extract	*Bacillus subtilis*	250 µg/mL	6.5	[89]
		*Bacillus cereus*	1000 µg/mL	-	
		*Staphylococcus epidermidis*	-	-	
		*Staphylococcus aureus*	-	-	
		Methicillin-resistant *Staphylococcus aureus*	-	6.5	
		*Enterobacter cloacae*	1000 µg/mL	7	
		*Enterobacter cloacae*	-	-	
		*Escherichia coli*	500 µg/mL	-	
		*Escherichia coli (Hemorrhagic, O157:H7)*	500 µg/mL	-	
		*Pseudomonas aeruginosa*	<50 µg/mL	-	
		*Proteus vulgaris*	250 µg/mL	-	
		*Salmonella typhimurium*	-	-	
		*Candida albicans*	-		
	Methanol extract	*Bacillus subtilis*	250 µg/mL	6.5	
		*Bacillus cereus*	500 µg/mL	-	
		*Staphylococcus epidermidis*	500 µg/mL	-	
		*Staphylococcus aureus*	500 µg/mL	-	
		Methicillin-resistant *Staphylococcus aureus*	-	7.5	
		*Enterobacter cloacae*	-	7	
		*Escherichia coli*	-	-	
		*Escherichia coli (Hemorrhagic, O157:H7)*	-	-	
		*Pseudomonas aeruginosa*	250 µg/mL	-	
		*Proteus vulgaris*	250 µg/mL	-	
		*Salmonella typhimurium*	-	-	
		*Candida albicans*	-	-	
	Dichloromethane extract	*Bacillus subtilis*	-	6.5	
		*Bacillus cereus*	-	-	
		*Staphylococcus epidermidis*	-	-	
		*Staphylococcus aureus*	-	-	
		Methicillin-resistant *Staphylococcus aureus*	-	-	
		*Enterobacter cloacae*	-	7	
		*Escherichia coli*	-	7	
		*Escherichia coli (Hemorrhagic, O157:H7)*	-	-	
		*Pseudomonas aeruginosa*	-	-	
		*Proteus vulgaris*	-	-	
		*Salmonella typhimurium*	-	-	
		*Candida albicans*	-	-	
*Codium intricatum*	Methanol extract	Methicillin-resistant *Staphylococcus aureus*	250 µg/mL	nd	[88]
		*Bacillus cereus*	500 µg/mL	nd	
		*Listeria monocytogenes*	500 µg/mL	nd	
		*Streptococcus mutans*	-	nd	
		*Pseudomonas aeruginosa*	-	nd	
		*Escherichia coli*	-	nd	
		*Enterobacter cloacae*	-	nd	
		*Salmonella typhimurium*	-	nd	
		*Aeromonas hydrophila*	-	nd	
*Codium tomentosum*	Methanolic extract	*Staphylococcus aureus*	nd	≥20	[90]
		*Escherichia coli*	nd	<10	
		*Klebliella pneumoniae*	nd	<10	
		*Enterobacter faecalis*	nd	<10	
*Enteromorpha compressa*	Ethanolic extract	*Salmonella* sp.	nd	15	[86]
		*Klebsiella* sp.	nd	10	
		*Proteus* sp.	nd	5	
		*Staphylococcus aureus*	nd	5	
*Enteromorpha* sp.	Methanol:acetone extract	*Pseudomonas aeruginosa*	150 g/mL	11 ± 0.2	[85]
		*Staphylococcus aureus*	100 g/mL	10 ± 0.2	
		*Escherichia coli*	100 g/mL	11 ± 0.2	
*Ulva fasciata*	Chloroform/methanol extract	*Escherichia coli*	640 µg/mL	7.33–10.67	[82]
		*Staphylococcus aureus*	>640 µg/mL	10.00–11.17	
*Ulva intestinalis*	Methanolic extract	*Vibrio fluvialis*	nd	7 ± 0.56	[79]
*Ulva intestinalis*	Benzene	*Escherichia coli*	nd	6	[81]
		*Klebsiella pneumoniae*	nd	-	
		*Pseudomonas aeruginosa*	nd	6	
		*Streptococcus pyogens*	nd	6	
		*Staphylococcus aureus*	nd	6	
	Butanol	*Escherichia coli*	nd	7	
		*Klebsiella pneumoniae*	nd	7	
		*Pseudomonas aeruginosa*	nd	6	
		*Streptococcus pyogens*	nd	7	
		*Staphylococcus aureus*	nd	6	
	Propanol	*Escherichia coli*	nd	6	
		*Klebsiella pneumoniae*	nd	-	
		*Pseudomonas aeruginosa*	nd	7	
		*Streptococcus pyogens*	nd	7	
		*Staphylococcus aureus*	nd	7	
	Acetone	*Escherichia coli*	nd	-	
		*Klebsiella pneumoniae*	nd	-	
		*Pseudomonas aeruginosa*	nd	-	
		*Streptococcus pyogens*	nd	-	
		*Staphylococcus aureus*	nd	-	
	Water	*Escherichia coli*	nd	6	
		*Klebsiella pneumoniae*	nd	10	
		*Pseudomonas aeruginosa*	nd	-	
		*Streptococcus pyogens*	nd	-	
		*Staphylococcus aureus*	nd	-	
*Ulva intestinalis*	Methanolic extract	*Escherichia coli*	nd	-	[84]
		*Klebsiella pneumoniae*	nd	-	
		*Proteus mirabilis*	nd	-	
		*Pseudomonas aeruginosa*	nd	-	
		*Salmonella typhi*	nd	-	
		*Vibrio alginolyticus*	nd	-	
		*Vibrio harveyi*	nd	-	
		*Vibrio parahaemolyticus*	nd	-	
		*Bacillus cereus*	1024 µg/mL	6.85 ± 0.17	
		*Enterobacter faecalis*	nd	-	
		*Listeria monocytogenes*		-	
		Methicillin-resistant *Staphylococcus aureus*	>1024 µg/mL	12.71 ± 0.98	
		*Staphylococcus aureus*	>1024 µg/mL	8.41 ± 0.56	
	Ethanolic extract	*Escherichia coli*	nd	-	
		*Klebsiella pneumoniae*	nd	-	
		*Proteus mirabilis*	nd	-	
		*Pseudomonas aeruginosa*	nd	-	
		*Salmonella typhi*	nd	-	
		*Vibrio alginolyticus*	nd	-	
		*Vibrio harveyi*	nd	-	
		*Vibrio parahaemolyticus*	nd	-	
		*Bacillus cereus*	nd	-	
		*Enterobacter faecalis*	nd	-	
		*Listeria monocytogenes*	>1024 µg/mL	7.96 ± 0.38	
		Methicillin-resistant *Staphylococcus aureus*	nd	-	
		*Staphylococcus aureus*	nd	-	
	Dichloromethane extract	*Escherichia coli*	nd	-	
		*Klebsiella pneumoniae*	nd	-	
		*Proteus mirabilis*	nd	-	
		*Pseudomonas aeruginosa*	nd	-	
		*Salmonella typhi*	nd	-	
		*Vibrio alginolyticus*	nd	-	
		*Vibrio harveyi*	nd	-	
		*Vibrio parahaemolyticus*	nd	-	
		*Bacillus cereus*	nd	-	
		*Enterobacter faecalis*	1024 µg/mL	-	
		*Listeria monocytogenes*	nd	9.89 ± 0.24	
		Methicillin-resistant *Staphylococcus aureus*	nd	-	
		*Staphylococcus aureus*	nd	-	
	Hexane extract	*Escherichia coli*	nd	-	
		*Klebsiella pneumoniae*	nd	-	
		*Proteus mirabilis*	nd	-	
		*Pseudomonas aeruginosa*	nd	-	
		*Salmonella typhi*	nd	-	
		*Vibrio alginolyticus*	nd	-	
		*Vibrio harveyi*	nd	-	
		*Vibrio parahaemolyticus*	nd	-	
		*Bacillus cereus*	256 µg/mL	7.28 ± 0.02	
		*Enterobacter faecalis*	nd	-	
		*Listeria monocytogenes*	1024 µg/mL	10.55 ± 0.29	
		Methicillin-resistant *Staphylococcus aureus*	256 µg/mL	16.4 ± 2.4	
		*Staphylococcus aureus*	256 µg/mL	12.13 ± 0.16	
*Ulva lactuca*	Benzene	*Escherichia coli*	nd	6	[81]
		*Klebsiella pneumoniae*	nd	6	
		*Pseudomonas aeruginosa*	nd	6	
		*Streptococcus pyogens*	nd	6	
		*Staphylococcus aureus*	nd	6	
	Butanol	*Escherichia coli*	nd	6	
		*Klebsiella pneumoniae*	nd	7	
		*Pseudomonas aeruginosa*	nd	-	
		*Streptococcus pyogens*	nd	-	
		*Staphylococcus aureus*	nd	8	
	Propanol	*Escherichia coli*	nd	6	
		*Klebsiella pneumoniae*	nd	6	
		*Pseudomonas aeruginosa*	nd	6	
		*Streptococcus pyogens*	nd	-	
		*Staphylococcus aureus*	nd	7	
	Acetone	*Escherichia coli*	nd	-	
		*Klebsiella pneumoniae*	nd	-	
		*Pseudomonas aeruginosa*	nd	8	
		*Streptococcus pyogens*	nd	-	
		*Staphylococcus aureus*	nd	-	
	Water	*Escherichia coli*	nd	-	
		*Klebsiella pneumoniae*	nd	-	
		*Pseudomonas aeruginosa*	nd	-	
		*Streptococcus pyogens*	nd	-	
		*Staphylococcus aureus*	nd	-	
*Ulva lactuca*	Chloroform/methanol extract	*Escherichia coli*	>640 µg/mL	7.33–10.67	[82]
		*Staphylococcus aureus*	640 µg/mL	10.00–11.17	
*Ulva lactuca*	N–hexane extract	*Staphylococcus aureus*	nd	10	[87]
		*Staphylococcus epidermidis*	nd	12	
		*Escherichia coli*	nd	11	
		*Pseudomonas aeruginosa*	nd	12	
	Chloroform extract	*Staphylococcus aureus*	nd	11	
		*Staphylococcus epidermidis*	nd	11	
		*Escherichia coli*	nd	11	
		*Pseudomonas aeruginosa*	nd	10	
	ethanol: water (1:1) extract	*Staphylococcus aureus*	nd	9	
		*Staphylococcus epidermidis*	nd	10	
		*Escherichia coli*	nd	9	
		*Pseudomonas aeruginosa*	nd	9	
*Ulva lactuca*	Polysaccharide (ulvan)	*Staphylococcus aureus*	-	-	[102]
		*Enterobacter faecalis*	-	-	
		*Bacillus subtilis*	12.50 ± 0.0 mg/mL	15 ± 0.50	
		*Listeria monocytogenes*	-	-	
		*Pseudomonas aeruginosa*	25.00 ± 0.0 mg/mL	12 ± 0.10	
		*Escherichia coli*	6.25 ± 0.0 mg/mL	11 ± 0.21	
		*Klebsiella pneumoniae*	6.25 ± 0.0 mg/mL	12 ± 0.00	
		*Bordetella pertussis*	-	-	
*Ulva reticulata*	Polysaccharide (ulvan)	*Bacillus cereus*	nd	-	[101]
		*Enterobacter faecalis*	nd	-	
		*Enterobacter cloacae*	nd	20.00 ± 1.00	
		*Staphylococcus aureus*	nd	-	
		*Escherichia coli*	nd	18 ± 0.5	
		*Pseudomonas aeruginosa*	nd	<18 ± 0.5	
		*Vibrio harveyi*	nd	-	
*Ulva* sp.	Methanolic extract	*Staphylococcus saprophyticus*	16 µg/mL	29 ± 0.592	[83]
		*Staphylococcus epidermidis*	4 µg/mL	26 ± 0.548	
		*Streptococcus agalactiae (group B)*	0.5 µg/mL	14 ± 0.592	
		*Enterobacter faecalis*	2 µg/mL	21 ± 0.592	
		*Stenotrophomonas maltophilia*	1 µg/mL	15 ± 0.592	
		*Salmonella enterica*	2 µg/mL	11 ± 0.592	
		*Shigella sonnei*	2 µg/mL	12 ± 0.592	
		*Pproteus vulgaris*	2 µg/mL	20 ± 0.592	
		*Pproteus mirabilis*	-	-	
		*Enterobacter cloacae*	-	-	
		*Haemophilus influenzae*	-	-	

## 5. Antimicrobial Mechanisms of Action of Seaweeds Compounds

Seaweeds have been used as traditional medicines and potential sources of new therapeutic agents for a long time. Research and development continue to carry on investigations of marine algae and their potential metabolites for human health. The development of seaweed is supported by the facts that seaweeds are easy to collect, easily cultivated, renewable, and they grow fast [103,104,105].

**Figure 4:**
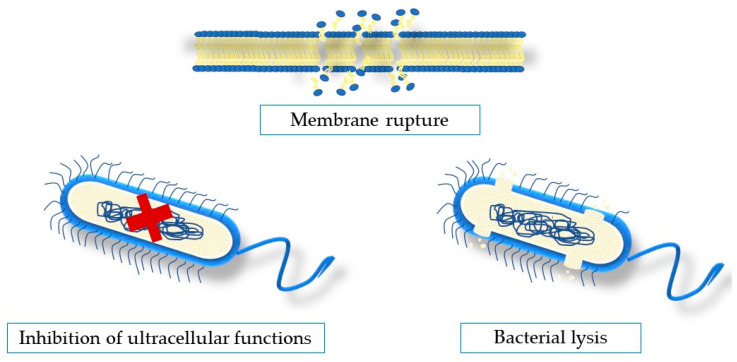
Possible mechanisms of antimicrobial activity exhibited by antibiotics and natural compounds.

The chemical profile of seaweeds and their therapeutic efficacy are largely influenced by a number of parameters such as species, physiological status, environmental variables (location, climate, temperature, salinity), growth conditions, environmental contamination, collecting period, thallus area, and epiphytic organisms [106,107]. Each seaweed species differs from another; thus, they all have their own unique characteristics. Moreover, differences may be due to different methods of extraction, solvents used in extraction, and different collecting seasons [108]. Macroalgae include a wide variety of taxonomic groups with different metabolites exhibiting biological properties, synthetized by seaweeds to overcome the harsh conditions in extreme environments [109]. Phenols, fatty acids, carbohydrates, proteins, and other minor chemicals have been identified as the chemical elements that give algae their antibacterial properties [110,111]. This section describes the possible mechanisms of antimicrobial activity of natural compounds (Figure 4).

Gram-negative bacteria are reportedly less sensitive to phenolic compounds’ bioactivity than Gram-positive bacteria, probably as a result of variations in cell wall composition [112]. The mechanisms of action of seaweed metabolites have not been clearly elucidated. According to earlier research, some phlorotannin antibacterial effect may be connected to their ability to integrate with microbial proteins, such as cell membranes and enzymes, and cause cell disintegration by inhibiting the oxidative phosphorylation pathway in microorganisms [113,114]. Different interactions, including those involving microbial membrane permeability, enzymatic inactivation, binding to surface membranes, and binding to surface sticky molecules, are thought to be involved in polyphenolic antibacterial bioactivity [115]. The study case of Hierholtzer et al. [116] displayed disrupted outer membranes, exo-polysaccharide coagulation, separation of the cytoplasmic membrane from the cell envelope, and "blebbing" and debris of phlorotannin-coagulated components, after the interaction between germs and phlorotannins from *Laminaria digitata*. The crucial phase involved with the bactericidal action of phlorotannin is the disruption of the cell envelopes, so the researchers concluded that this disruption is linked with the level of polymerization of the compounds [116].

Phlorotannins are thought to cause cell lysis as a result of their antibacterial activity. The inclusion of hydroxyl groups in the phlorotannin molecule, which may bind to amide groups in the bacterial proteins, can further strengthen these interactions [117,118]. The -NH groups of bacterial proteins presumably interact with aromatic rings and -OH groups of the phloroglucinol monomer through hydrophobic interactions and H-bonds [112,117,119,120]. Depending on the species and time of year they are harvested, seaweeds are a promising source of proteins. Brown algae usually contain a low quantity of proteins (3–15% DW), while an intermediate quantity is found in green algae (9–33% DW), and a high quantity in red seaweeds (47% DW) [121]. Relevant proteins with antibacterial activity are lectins or agglutinins; these substances are glycoproteins that can detect free sugars or glycoconjugates and interact with them in a reversible manner without altering their structural integrity [122]. Lectins have been investigated as antibacterial, anti-inflammatory, anti-adhesion, anti-cancer, and antimicrobial agents [122].

Fatty acids and monoglycerides that interact with bacterial cell membranes and have antibacterial activity are identified as antimicrobial lipids [123]. Myristic, palmitic, oleic, and eicosapentaenoic acids, which are linked to the antibacterial characteristics of algae, are the most prevalent saturated and unsaturated fatty acids found in seaweed [124]. Seaweeds typically have low levels of lipids (0.4 to 5% DW basis); however, they do include important lipids including glycolipids and polyunsaturated fatty acids such as omega-3 that have important biological features and health advantages [125]. Additionally, a range of secondary metabolites and chemicals found in essential oils from various seaweeds are known to slow down or prevent the growth of bacteria, yeast, and molds [126,127,128,129,130]. It appears that the mechanism of action is due to the membrane-lytic response of fatty acids, which results in membrane instability and defect formation and, as result, inhibits cell development (bacteriostatic action) or even causes cell death. These molecules can also interfere with two vital cellular processes for energy production: oxidative phosphorylation (by reducing the membrane potential and proton gradient) and the electron transport chain (by attaching to electron carriers or changing the integrity of the membranes). Fatty acids can also directly disrupt membrane enzymes and obstruct the cell’s ability to absorb nutrients [123].

Macroalgal polysaccharides’ antibacterial mechanisms of action are still not completely understood. According to Zhao et al. [131], the antibacterial properties of fucoidan may be connected to the amounts of glucuronic and sulfuric acids that are released when the molecules depolymerize. The results announce that fucoidans have the ability to attach to the proteins that assemble bacterial membranes, leading to membrane cell breakdown and cell death [131]. Compared to Gram-positive bacteria, Gram-negative bacteria appear to be less responsive to fucoidans antibacterial properties. This phenomenon was explained by the existence of cell wall elements that could serve as a barrier to the antibacterial effects of fucoidans [131]. The study of He et al. [132] suggested that the cell wall, cytoplasmic membranes, and DNA may be the primary targets of antibacterial polysaccharides. These results came out from investigating the antibacterial activities of seaweed polysaccharides against bacteria that cause food spoilage and food poisoning, including *Candida utilis*, *S. aureus*, *B. subtilis*, *L. monocytogenes*, and *E. coli* [132].

Among minor compounds, sterols are the most diverse in nature and have a variety of features. The antibacterial properties of the sterol 24-propylidene cholest-5-en-3-ol isolated from *L. papillosa* against a range of bacteria were determined by Kavita et al. [133]. Similar to this, earlier studies on macroalgal sterols noted their potent antibacterial properties [134,135,136]. Previous research revealed that the presence of sterols may impact the morphological responses in the cell membranes when induced by antimicrobial lipids, and therefore lead to the disruption of the bacterial membranes. However, the antimicrobial mechanisms of action of these substances are still not entirely elucidated and further studies need to be carried out [137].

## 6. Conclusions

As the present review shows, various plants and macroalgae developed several chemicals with antimicrobial activities. Moreover, due to the therapeutic efficacy of their active ingredients, medicinal plants play a significant part in the restoration of damage caused by microbial infection. Many plant products, including the entire plant, medicinal volatile oils, extracts, etc., have been used as natural antibiotics for treating burns, respiratory tract infections, boosting the immune system, lowering blood pressure, and other conditions wherein microbes can infect and thrive inside or outside of the body once our immune system is weak. Natural antibiotics are herbs and spices that can be found in nature which have qualities that stop pathogens from functioning and spreading, with less severe consequences than those of conventional antibiotics.

The necessity to replace synthetic antibiotic compounds with natural ones is due to the action of synthetic antibiotics during their assumption; even though antibiotics kill infectious bacteria, they also cause a significant amount of side effects by disturbing natural functions of the body and destroying intestinal flora bacteria [138,139,140]. Herbal antibiotics destroy bacteria as well, purify the blood, boost the immune system, and improve organ system functions. They function by killing microorganisms and correcting body imbalances. Additionally, most herbal antibiotics do not develop drug resistance and do not have the bactericidal effect against beneficial bacteria which live in our body [141].

It is misleading to say that herbal medicines have no toxic effects at all, or any side effects. There are cases where the uptake of large amounts of natural products in high concentrations of herbal complements may badly influence our organisms [142,143]. For example, taking garlic in high concentrations may enhance the risk of bleeding, so it is not suggested for people having surgery or taking blood thinners [144]. Therefore, to avoid unpleasant consequences, it is important to take medicine, herbal or synthetic, in proper dosage and proper course of treatment.

Although there is evidence that natural plants and macroalgae extracts are a source of antimicrobial chemicals, further research is required to understand the mechanisms by which plant and macroalgal substances affect cells, as well as clinical trials. The pharmacochemical profiles, pharmacological outcomes, and evaluations of the inclusion of these natural chemicals in new antibiotics must be gathered. As we see through this manuscript, the different types of extraction and solvents used influence the antimicrobial activity. To reduce the occurrences of drug-resistant microorganisms, it is vital to go further in the development of herbal medicines and find the best extraction methodology with remunerative costs and yield. The use of herbal drugs as medicine has the potential to provide biocompatible, less expensive, and effective herbal solutions and will increase the probability of the discovery of new natural antibiotics. Further and more in-depth studies are required to build an understanding on the mechanism of action, identify, and isolate the specific compounds responsible for the desired effects and use them as an alternative to synthetic drugs.

## Data Availability

Not applicable.
